# Intratumoral and peritumoral multiparametric MRI-based radiomics nomogram for preoperative risk stratification in patients with endometrial cancer

**DOI:** 10.3389/fonc.2025.1572784

**Published:** 2025-08-26

**Authors:** Bin Yan, Tingting Zhao, Ying Deng, Jianrong Lu, Guoqing Wang

**Affiliations:** ^1^ Department of Radiology, Shaanxi Provincial Tumor Hospital, Xi’an, China; ^2^ Department of Medical Imaging, First Affiliated Hospital of Xi’an Jiaotong University, Xi’an, Shaanxi, China; ^3^ Department of Pathology, Shaanxi Provincial Tumor Hospital, Xi’an, China; ^4^ Department of Gynecologic Tumor, Shaanxi Provincial Tumor Hospital, Xi’an,, China

**Keywords:** endometrial cancer, risk stratification, peritumoral, magnetic resonance imaging, radiomics

## Abstract

**Introduction:**

Achieving accurate preoperative risk stratification for endometrial cancer (EC) is challenging due to the need for histopathology to obtain the necessary parameters. This study aimed to establish and validate a multiparametric magnetic resonance imaging (MRI) radiomics nomogram that incorporates the peritumoral region for preoperative risk stratification in EC patients.

**Methods:**

Three-hundred seventy-four women with histologically confirmed EC were divided into training (1.5-T MRI, n=163), test (1.5-T MRI, n=70), and independent validation (3.0-T MRI, n=141) cohorts. As per the guidelines of the European Society of Medical Oncology, patients were categorized into four risk groups: low, intermediate, high-intermediate, and high. Binary classification models were subsequently constructed to distinguish between low- and non-low-risk individuals. Radiomic features were extracted from intra- and peritumoral regions via T2-weighted imaging (T2WI) and apparent diffusion coefficient (ADC) maps. Feature selection was carried out via univariate analysis, least absolute shrinkage and selection operator (LASSO) regression, and multivariate logistic regression. A radiomic model (radscore) was established using the selected features. A nomogram combining the radscore and most predictive clinical parameters was developed. Decision curve analysis (DCA) and the net reclassification index (NRI) were used to assess the clinical benefit of the nomogram.

**Results:**

Nine radiomic features were selected from intra- and peritumoral regions on ADC maps and T2W images. The nomogram combining the radscore, age, maximum anteroposterior tumor diameter on sagittal T2WI (APsag), and the tumor area ratio (TAR), achieved the highest area under the curve (AUC) values across all cohorts (training: 0.949, test: 0.947, independent validation: 0.909). The nomogram demonstrated superior performance compared to the radscore (AUCtraining = 0.929, AUCtest = 0.917, and AUCindependent validation = 0.813) alone and the clinical model (AUCtraining = 0.855, AUCtest = 0.845, and AUCindependent validation = 0.842). DCA and the NRI demonstrated that the nomogram achieved greater diagnostic performance and net clinical benefits than did the radscore alone.

**Conclusion:**

The developed MRI radiomics nomogram achieved high diagnostic performance in classifying low- and non-low-risk EC preoperatively. This tool could provide valuable support for therapeutic decision-making and demonstrates robustness across various field strength data, increasing its potential for widespread clinical application.

## 1 Introduction

Endometrial cancer (EC) is the sixth most common cancer and the fourth leading cause of cancer-related death among women globally (1). Its effective management and prognosis depend on accurate risk stratification, which can guide treatment decision-making, especially regarding lymphadenectomy (2). The European Society for Medical Oncology (ESMO) classifies EC into four risk groups according to histological grade, cell type, depth of myometrial invasion (MI), lymphovascular space invasion (LVSI), extrauterine invasion, nodal involvement, and molecular subgroups (3). Low-risk EC includes LVSI-negative International Federation of Gynecology and Obstetrics (FIGO) stage IA endometrioid endometrial carcinoma (EEC), grades 1-2; intermediate-risk EC includes LSI-negative, FIGO stage IB EEC, grades 1-2; high-intermediate-risk EC includes FGO stage IA EEC grade 3 (with or without LVSI) or LVSI-positive stage IA/IB EEC, grades 1-2; and high-risk EC consists of FIGO stage IB EEC grade 3 (with or without LVSI), FIGO stages ≥ II EEC, and non-EEC tumors (4). Low-risk EC is treated with total hysterectomy and bilateral salpingo-oophorectomy (THBSO), whereas high-risk EC may require THBSO, lymphadenectomy, or adjuvant therapy (4). Thus, precise preoperative risk stratification is crucial for determining the appropriate individualized treatment.

Magnetic resonance imaging (MRI), which is highly specific for assessing deep myometrial invasion (DMI) in EC, is recommended for the preoperative staging of EC patients (5). Diffusion-weighted imaging (DWI) provides *in vivo* insights into tissue cell density and has proven valuable in predicting tumor grade, DMI, and LVSI in EC patients (6). Various tumor morphological parameters (such as tumor size (TS, tumor maximum diameter), the tumor volume ratio (TVR), the tumor area ratio (TAR), and the maximum anteroposterior tumor diameter on sagittal T2-weighted images (APsag)) evaluated via MR images are correlated with DMI, tumor grade, lymph node metastasis (LNM), and the LVSI status in EC patients (7–10). These results suggest that tumor morphological indices could be leveraged for predicting ESMO classification. A recent study developed a nomogram from tumor morphological parameters that predicted non-low-risk EEC in patients, performing well in a cross-field MR cohort (area under the curve (AUC): 0.856 in the training set, 0.849 in the validation set). In addition to tumor morphology, clinical factors such as age and serum CA125 levels are linked to EC risk.

Radiomics, an emerging technology, can reveal imaging biomarkers that are not easily detectable by the human eye, thereby enhancing risk stratification with additional information (11). Numerous studies have highlighted the utility of radiomics—which involves imaging methods such as ultrasound, computed tomography (CT), and MRI—in different facets of EC management, including forecasting lymph node metastasis (LNM) (12), assessing MI (13), evaluating tumor grade (14), predicting high-risk EC (15), tumor recurrence (16), and survival (17). Recent studies have underscored the importance of examining not only intratumoral but also peritumoral regions in radiomic analysis (12). The tumor microenvironment, which includes the peritumoral area, plays a critical role in cancer progression and metastasis. Therefore, the incorporation of features from both intratumoral and peritumoral regions may yield a more comprehensive assessment of tumor biology and increase the accuracy of risk prediction.

The integration of radiomic features with clinical and conventional imaging parameters has facilitated the creation of radiomic nomograms, demonstrating potential in enhancing diagnostic precision and risk prognostication across various cancer types (18). In the context of EC, radiomic nomograms have been investigated for their utility in predicting LVSI (19), evaluating DMI (20), and assessing overall survival (21). Few studies have employed radiomics to perform preoperative risk stratification in patients with EC (15), focusing solely on the use of intratumoral radiomic data. To our knowledge, no study has attempted to construct a risk stratification model that incorporates peritumoral radiomic information.

## 2 Materials and methods

### 2.1 Patients

Approval for this retrospective study was granted by the institutional review board, and the necessity for informed consent was waived. A total of 461 patients, all with confirmed EC through postoperative histology from June 2015 to July 2022, were enrolled in the study following preoperative MR examination. The study included patients who met the following criteria: (1) confirmation of EC through histopathology; and (2) availability of clinical and histopathological characteristics, such as age, histopathological type, tumor grade, MI depth and cervical stromal invasion (CSI), EI, LVSI and postoperative lymph node status. The exclusion criteria were as follows: (1) absence of total hysterectomy within 2 weeks post-MRI; (2) prior chemoradiation treatment; (3) tumors too small for MRI detection; (4) images with evident motion artifacts; (5) MRI contraindications; (6) incomplete clinical data; and (7) concurrent EC with other malignancies. A total of 374 patients, with a mean age of 54.3 ± 8.1 years, were included in the study after 87 patients were excluded. Surgical staging for all patients consisted of total hysterectomy with bilateral salpingo-oophorectomy and lymph node assessment, which included pelvic lymphadenectomy and paraaortic lymphadenectomy.

**Figure 1:**
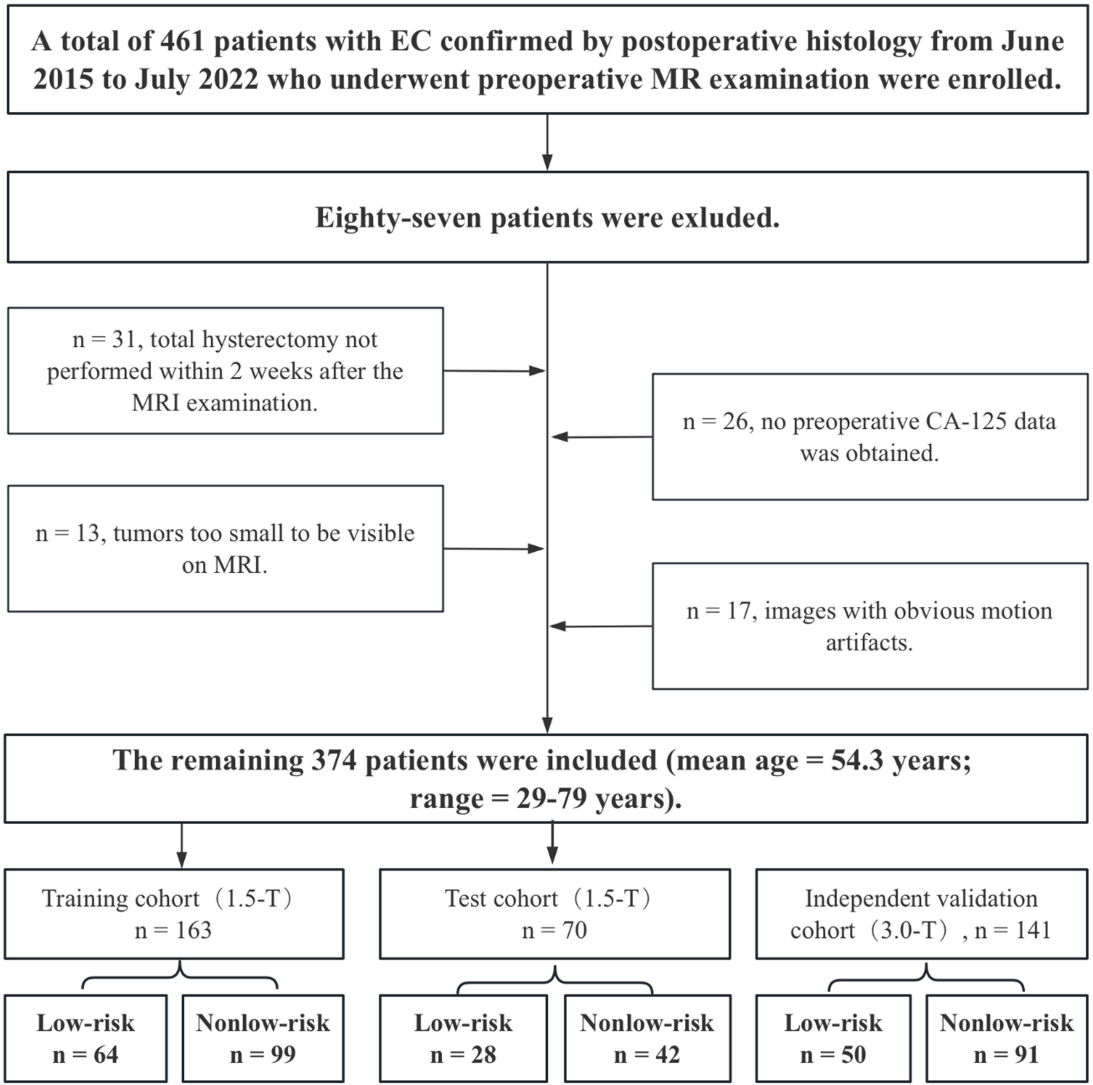
Flow chart showing the selection of the study population, the exclusion criteria, and the grouping approach.

The 1.5-T MRI dataset (n = 233) was divided into training (n = 163) and testing (n = 70) sets at a 7:3 ratio for selecting features and building prediction models. To improve the model’s generalizability, a 3.0-T dataset (n = 141) was assigned as an independent validation cohort. Importantly, the independent validation cohort was not part of the model training and testing phases. **Figure 1** displays a flow chart outlining the patient demographics and exclusion criteria.

### 2.2 Risk stratification of patients with EC

According to ESMO guidelines (22), individuals with EC were classified into four risk groups: low, intermediate, high-intermediate, and high. Given that lymphadenectomy is not recommended for low-risk ECs, we opted to develop a binary classification model to differentiate between patients in the low-risk and non-low-risk groups.

### 2.3 MRI protocol

MRI scans were conducted using either a 1.5-T (EXCELART VantageTM powered by Atlas, Canon Medical Systems Corp., Tochigi, Japan) or a 3.0-T (Siemens Magnetom Skyra, Erlangen, Germany) scanner equipped with an 8-channel phased-array abdominal coil. Prior to scanning, a 20-mg injection of raceanisodamine hydrochloride (Hangzhou People’s Livelihood Pharmaceutical Co.) was given intravenously to minimize artifacts caused by intestinal motility. All MRI sequences were obtained following the standard protocol, with specific details provided in **Supplementary Table 1**. Diffusion-weight imaging (DWI) uses b values of 0 and 650 s/mm^2^ for the 1.5-T scanner and b values of 0 and 1000 s/mm^2^ for the 3.0-T scanner. ADC maps were automatically generated via a postprocessing workstation.

### 2.4 Radiomic features

**Figure 2:**
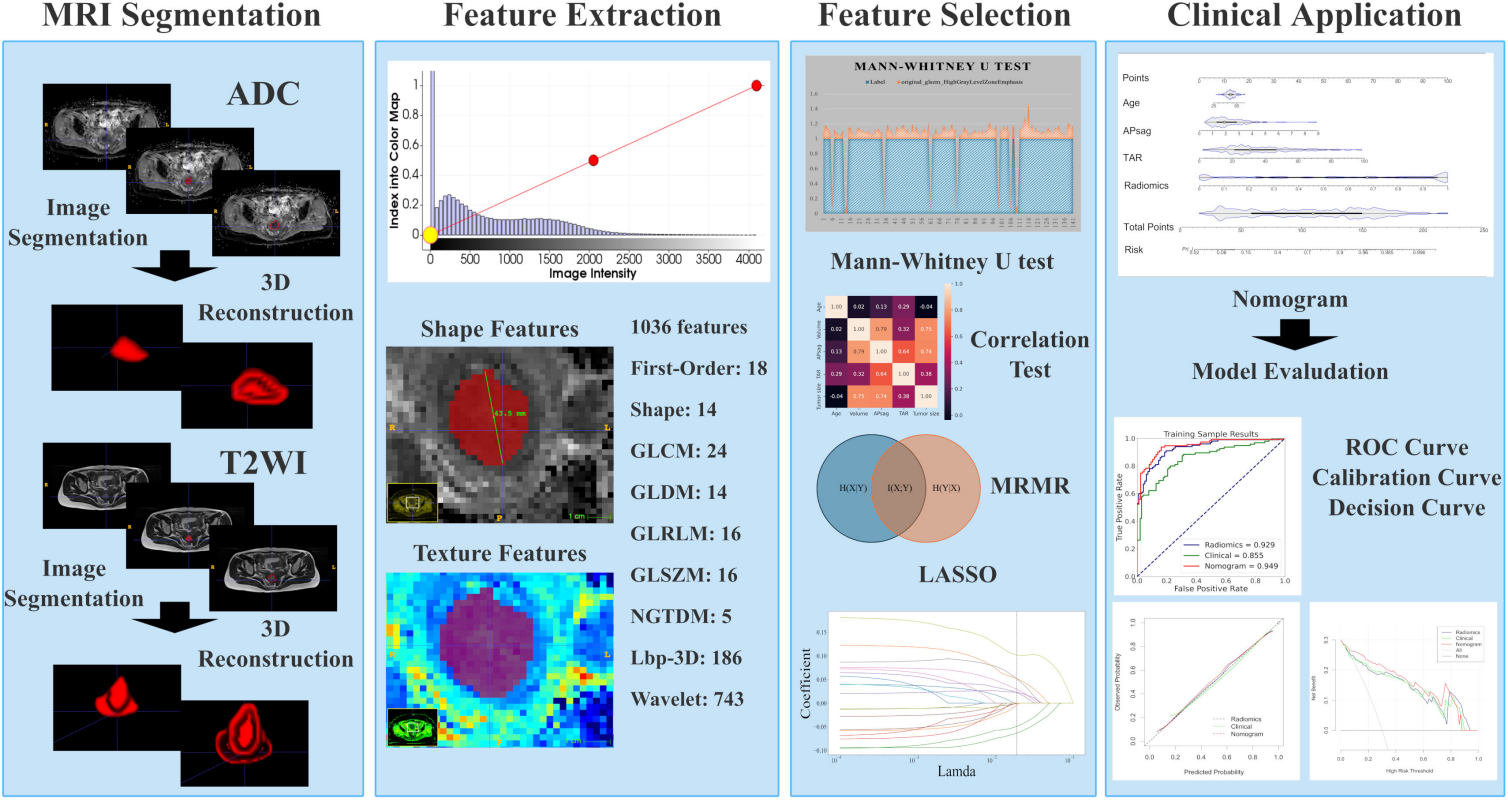
Radiomic workflow. First, 3D VOIs for both the intra-tumoral and peritumoral areas were manually segmented. Second, radiomic features were extracted. Third, feature selection was carried out through the Mann-Whitney U test, the minimum redundancy maximum relevance (mRMR) algorithm, correlation tests, and least absolute shrinkage and selection operator (LASSO) regression. Fourth, the model was developed in the form of a nomogram. Finally, the diagnostic performance was evaluated via receiver operating characteristic (ROC) curve, calibration curve, and decision curve analyses.

Image preprocessing was conducted following a standard workflow detailed in the **Supplementary Materials**. Two experienced radiologists (T.Z. and B.Y.) were responsible for image segmentation. After one month, 100 patients were randomly chosen for tumor segmentation by a different radiologist (Y.D.) to evaluate interreader reliability. More information on lesion segmentation can be found in the **Supplementary Materials**. Manual whole-tumor segmentation was performed via 3D Slicer software (version 4.10.2; https://download.slicer.org/) on axial T2WI and apparent ADC maps. Three volumes of interest (VOIs) were selected, as shown in **Figure 2**. The first VOI consisted of the intratumoral region, where regions of hemorrhage and necrosis were delineated along the edge of the lesion slice-by-slice, while normal anatomical structures were avoided. These ROIs were combined to create a three-dimensional (3D) VOI. The second VOI was the peritumoral margin, which was generated by automatically expanding the tumor contour by 3 mm. If the dilated VOIs extended beyond the uterus, manual corrections were made to align the boundaries with the uterus edge. Additionally, any other lesions in the myometrium were manually identified to not be included in the dilated VOI. The peritumoral region was defined as the dilated VOI minus the tumor VOI. The third VOI consisted of a combination of the intra- and peritumoral regions.

An artificial intelligence kit (AK, Version 3.3.0, GE Healthcare) software was used to preprocess the images, and features were extracted. Radiomics features, which include first-order, shape-based, and texture features, were calculated for each VOI. The methodology for obtaining the radiomic parameters is illustrated in **Figure 2**.

### 2.5 Tumor morphological parameter measurements

**Figure 3:**
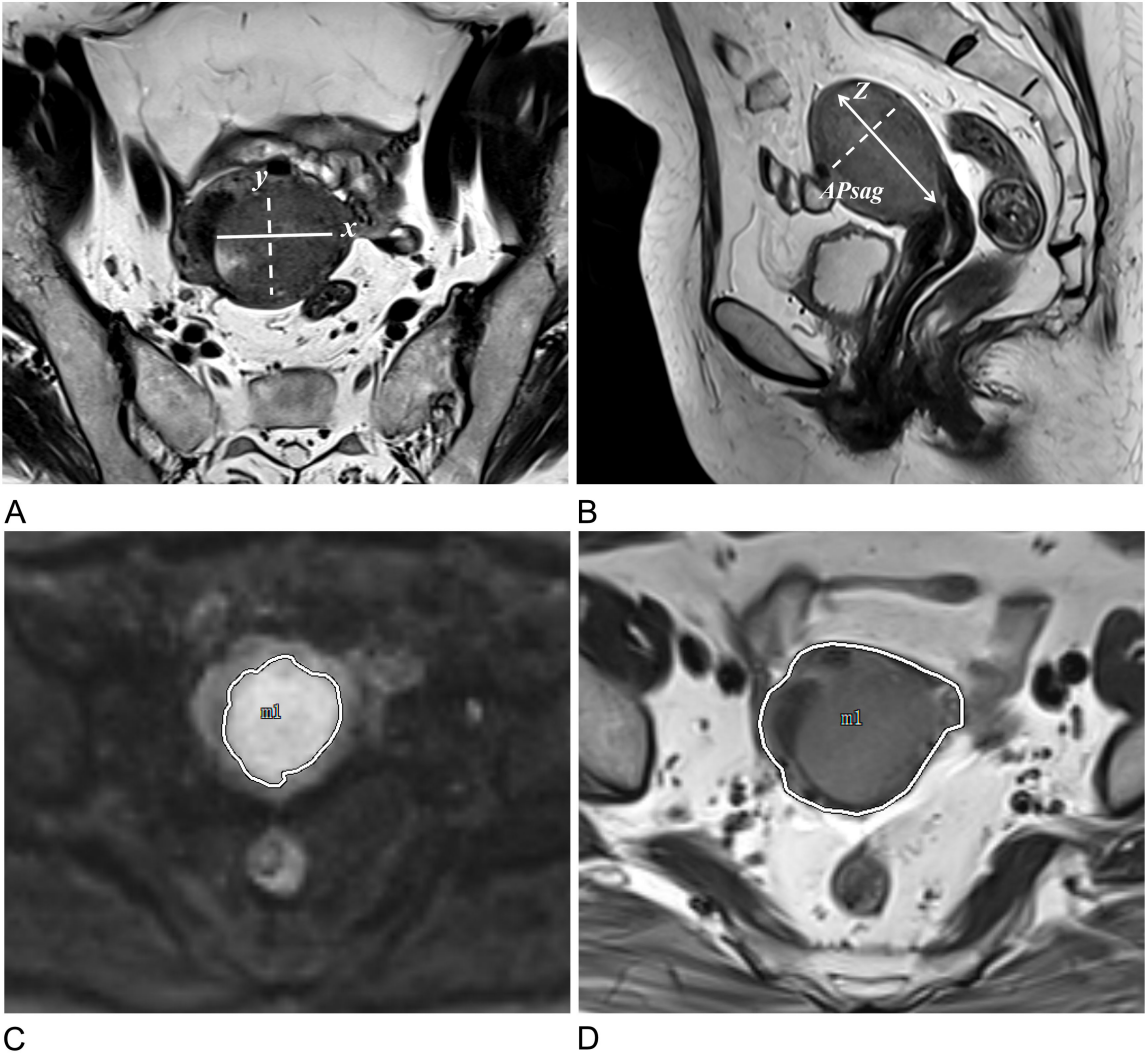
Approaches to measuring tumor morphology parameters. **(A)** Measurements of the tumor’s maximum transverse (x) and anteroposterior (y) diameters were conducted on oblique axial T2W images. **(B)** The tumor’s maximum craniocaudal (z) and anteroposterior (APsag) diameters were determined on sagittal T2W images. **(C)** The tumor border on the DW image is delineated by the white solid line. **(D)** The uterine border on the axial T2W image is outlined by the white solid line.

Since TS, tumor volume (TV), TAR, and APsag have been linked to LVSI, tumor grade, and DMI in EC, these factors were assessed as follows (7, 9, 11, 23). TS was measured in three dimensions: the transverse (x) and anteroposterior (y) diameters were measured on oblique axial T2-weighted images, and the craniocaudal (z) diameter was measured on sagittal T2-weighted images (**Figures 3A, B**). APsag was measured on sagittal T2-weighted images (**Figure 3B**). According to the protocol in a previous study (9), the TAR was calculated (**Figures 3C, D**) via the following formula: TAR = (tumor area/uterus area) × 100%.

### 2.6 Statistical analysis

The statistical analysis was carried out via the R language (version 4.2.0, https://www.r-project.org) and the Python language (version 3.9, https://www.python.org). A binary classification model was created to forecast the risk categorization as either “low-risk” or “non-low-risk.” Clinical data were analyzed through both univariate and multivariate logistic regression (LR) analyses for filtration, with radiomic features being examined via t tests, Fisher’s exact tests, chi-square tests, and, when relevant, the Mann–Whitney U test. Statistical significance was defined as P <0.05. The most important radiomic features were identified via least absolute shrinkage and selection operator (LASSO) regression, with LR used for training the prediction models and creating a nomogram that incorporated clinical and tumor morphological parameters, along with the radiomic score (radscore). The performance of the predictive models was assessed using the AUC values in the training, test, and independent validation cohorts. The independent validation cohort was utilized solely for evaluating model performance. On the basis of prior research (24), the sample size was calculated to ensure a minimum of 10 events per variable in the multivariate LR model. Radiomic features were standardized via the standard scalar method before data modeling. Intraclass correlation coefficients (ICCs) were computed to evaluate the consistency among readers in assessing MR morphological and radiomics features, with an ICC value above 0.8 indicating nearly perfect agreement.

The evaluation of the prediction model was conducted via receiver operating characteristic (ROC) curve analysis, whereas the calibration curve was assessed via the Hosmer–Lemeshow (HL) test. A P value greater than 0.05 indicated satisfactory predictive performance. Decision curve analysis (DCA) was employed to compare the net benefits of the clinical models and radiomics nomogram models, with ROC curve cutoff values determined by the maximum Youden index. The AUC, accuracy (ACC), sensitivity (SEN), and specificity (SPE), positive predictive value (PPV), and negative predictive value (NPV) were subsequently calculated.

## 3 Results

### 3.1 Histopathologic and MRI morphological findings

The histopathological and MRI morphological findings of the EC patients in the training, test, and independent validation cohorts are summarized in **Table 1**. Among the 374 EC patients, 233 patients underwent 1.5 T MRI scans, and 141 patents underwent 3.0 T MRI scans. Among these patients, 142 patients were classified as low risk, representing 38.0% of the total, and 232 patients were non-low-risk patients. The percentages of non-low-risk patients in the training (60.7%, 99/163) and (60.0%, 40/70) testing sets were comparable.

**Table 1:** Clinical and morphological parameters and histopathological characteristics of 374 patients.

Characteristic	Training cohort (1.5-T, n = 163)		*P*	Test cohort (1.5-T, n = 70)		Independent-validation cohort (3.0-T, n = 141)	
	Low-risk (n = 64)	Nonlow-risk (n = 99)		Low-risk (n = 28)	Nonlow-risk (n = 42)	Low-risk (n = 50)	Nonlow-risk (n = 91)
Age, years	52.3 ± 7.0	56.1 ± 8.1	**0.003**	50.5 ± 7.0	56.3 ± 7.9	49.9 ± 7.7	56.4 ± 7.7
EEC	64	80		28	38	50	74
Non-EEC		19			4		17
Histological grade							
Grade 1 (G1)	6	1		4		9	4
Grade 2 (G2)	58	46		24	23	41	46
Grade 3 (G3)		52			19		41
MI							
Superficial	64	44		28	17	50	38
Deep		55			25		53
CSI							
Yes		36			21		27
No	64	63		28	21	50	64
LVSI							
Present		43			28		42
Absent	64	56		28	14	50	49
LNM							
PLN		9					1
PALN		4					4
Tumor volume, cm^3^	7.709 ± 13.721	28.752 ± 54.362	**0.000**	6.966 5.752	28.355 ± 36.165	10.433 ± 16.144	32.696 ± 92.806
Tumor size, cm	3.426 ± 1.604	4.866 ± 2.525	**0.000**	3.350± 1.207	5.288 ± 2.259	3.758 ± 1.877	4.893 ± 2.683
APsag, cm	1.406 ± 0.674	2.626 ± 1.366	**0.000**	1.484 ± 0.597	2.835 ± 1.545	1.318 ± 0.777	2.552 ± 1.567
TAR, %	23.041 ± 10.977	41.278 ± 18.663	**0.000**	26.804 ± 14.517	50.396 ± 23.153	20.325 ± 9.733	41.044 ± 21.279
FIGO							
I	64	53		28	17		50
I a	64	24		28	6	50	23
I b		29			11		27
II		24			13		13
III		21			10		26
III a		8			1		8
III b							1
III c1		9			6		12
III c2		4			3		5
IV		1			2		2
IV a		1			1		2
IV b					1		
Risk stratification							
Low	64			28		50	
Intermediate		6			4		11
High-intermediate		24			8		23
High		69			30		57

EEC, endometrioid endometrial carcinoma; Non-EEC, Non-endometrioid endometrial carcinoma; LVSI, lymphovascular space invasion; G1, well differentiated; G2, moderately differentiated; G3, poorly differentiated; MI, myometrial invasion; CSI, cervical stromal invasion; LNM, lymph node metastasis; PLN, pelvic lymph node; PALN, para-aortic lymph node; FIGO, International Federation of Obstetrics and Gynecology. The words in bold indicate the indicators that have statistical differences.

### 3.2 Radiomics feature extraction, selection, and interreader reliability

Each VOI yielded 1036 features, including ADCintratumoral, ADCperitumoral, T2WIintratumoral, and T2WIperitumoral features. After feature selection, nine features were identified to discriminate between low- and non-low-risk patients with EC, collectively forming the radscore presented in **Table 2**. Additional information on the feature extraction process can be found in the **Supplementary Tables 2**, **3**. The features in the training and test sets were not significantly different (**Table 3**).

**Table 2:** Features forming the radscore using the logistic regression classifier.

Imaging	Region	Feature
ADC mapping	Intratumoral	original_shape_LeastAxisLength
ADC mapping	Intratumoral	wavelet-HHH_firstorder_Skewness
ADC mapping	Peritumoral	wavelet-LLH_glcm_MCC
ADC mapping	Peritumoral	original_shape_LeastAxisLength
T2-weighted imaging	Intratumoral	original_glcm_Contrast
T2-weighted imaging	Intratumoral	wavelet-HHL_firstorder_Kurtosis
T2-weighted imaging	Intratumoral	wavelet-HLH_firstorder_Skewness
T2-weighted imaging	Peritumoral	original_shape_LeastAxisLength
T2-weighted imaging	Peritumoral	original_glcm_ClusterShade

ADC, apparent diffusion coefficient.

**Table 3:** Comparison of the radiomic, clinical and morphological features in the training and test sets.

Features	*P value*
Age	0.398
APsag	0.867
TAR	0.542
original_shape_LeastAxisLength (ADCintratumoral)	0.724
wavelet-HHH_firstorder_Skewness (ADCintratumoral)	0.806
wavelet-LLH_glcm_MCC (ADCperitumoral)	0.547
original_shape_LeastAxisLength (ADCperitumoral)	0.728
original_glcm_Contrast (T2intratumoral)	0.676
wavelet-HHL_firstorder_Kurtosis (T2intratumoral)	0.528
wavelet-HLH_firstorder_Skewness (T2intratumoral)	0.463
original_shape_LeastAxisLength (T2peritumoral)	0.732
original_glcm_ClusterShade (T2peritumoral)	0.912

The ICCs for all the morphological parameters and radiomic features indicated outstanding interreader reliability, ranging from 0.902 to 0.997. This strong agreement ensures the model’s consistency for future clinical use. See **Supplementary Tables 4**-**6** for further details.

### 3.3 Clinical model development and performance

Univariate analysis revealed that age, TS, TV, APsag, and TAR were significantly different between low- and non-low-risk patients (all P < 0.05). Multivariate binary LR analysis revealed that age, APsag, and TAR were independent predictors for classifying low- and non-low-risk patients with EC (all P < 0.05, **Table 4**). The AUCs of the clinical model for classifying low-risk and non-low-risk EC patients were 0.855 (95% CI: 0.806–0.887; SEN: 73.7%, SPE: 79.4%) with the training cohort, 0.845 (95% CI: 0.799–0.873; SEN: 79.5%, SPE: 84.8%) with the test cohort, and 0.842 (95% CI: 0.805–0.886; SEN: 70.3%, SPE: 84.0%) with the independent validation cohort.

**Table 4:** Logistic regression analysis results in classifying patients with EC into the low-risk and nonlow-risk groups with the clinical model constructed from the training cohort data.

Parameters	Univariate			Multivariate		
	OR	95% CI	*p*	OR	95% CI	*p*
Age (years)	1.078	1.039-1.121	**<0.001**	1.066	1.019-1.120	**0.007**
Tumor volume (cm^3^)	1.077	1.045-1.116	**<0.001**	0.993	0.971-1.032	0.682
Tumor size (cm)	1.576	1.332-1.903	**<0.001**	1.084	0.836-1.415	0.548
APsag (cm)	4.723	3.084-7.693	**<0.001**	2.927	1.507-5.953	**0.002**
TAR (%)	1.080	1.056-1.107	**<0.001**	1.034	1.006-1.066	**0.019**

CI, confidence interval; OR, odds ratio. The words in bold indicate the indicators that have statistical differences.

### 3.4 Radiomics model development and performance

The performance of the radiomic models for different VOIs is shown in **Table 5**. The combined feature model exhibited better predictive performance than did the single-region prediction model with both the training and testing cohorts, regardless of ADC maps or T2W images (**Table 5**). Additionally, the combined feature model achieved moderate predictive accuracy with the independent validation cohort (**Table 6**).

**Table 5:** Comparison of two types of imaging sequence features from different VOIs in the training and test cohorts.

Imaging sequence	Training cohort						Testing cohort					
	Intratumoral		Peritumoral		Combined regions		Intratumoral		Peritumoral		Combined regions	
	AUC (95%CI)	ACC%	AUC (95%CI)	ACC %	AUC (95%CI)	ACC %,	AUC (95%CI)	ACC %	AUC (95%CI)	ACC %	AUC (95%CI)	ACC %
ADC	0.836 (0.788-0.864)	77.2	0.797 (0.758-0.826)	74.6	0.850 (0.812-0.887)	78.2	0.803 (0.766-0.836)	77.7	0.763 (0.725-0.814)	67.1	0.848 (0.812-0.879)	72.9
T2WI	0.852 (0.822-0.896)	74.6	0.842 (0.800-0.873)	75.6	0.887 (0.854-0.923)	81.2	0.851 (0.817-0.886)	74.1	0.827 (0.766-0.857)	71.8	0.858 (0.824-0.903)	77.7

ADC, apparent diffusion coefficient; AUC, area under the curve; ACC, accuracy; CI, confidence interval.

**Table 6:** Comparison of two types of imaging sequence features from different VOIs in the independent validation cohort.

Imaging sequence	Independent-validation cohort (3.0-T MRI, n = 141)					
	Intratumoral		Peritumoral		Combined regions	
	AUC (95%CI)	ACC %	AUC (95%CI)	ACC %	AUC (95%CI)	ACC %,
ADC	0.729 (0.687-0.765)	69.5	0.732 (0.690-0.768)	69.5	0.719 (0.675-0.766)	69.5
T2WI	0.810 (0.773-0.847)	72.3	0.755 (0.722-0.804)	67.4	0.810 (0.766-0.847)	71.6

ADC, apparent diffusion coefficient; AUC, area under the curve; ACC, accuracy; CI, confidence interval.

To improve prediction accuracy, we opted to construct a prediction model by merging VOIs from different sequences (**Table 7**). The hybrid features derived from this model (ADCintratumoral, ADCperitumoral, T2WIintratumoral, and T2WIperitumoral) led to superior classification performance. The model achieved an AUC of 0.929 and an accuracy of 85.3% with the training set, an AUC of 0.917 and an accuracy of 81.2% with the test set, and an AUC of 0.813 and an accuracy of 73.1% with the independent validation set. In this study, the radscore was composed of 9 features selected from the hybrid feature model (**Table 2**).

**Table 7:** Combination of features from different imaging sequences and VOIs for classifying patients with EC into the low-risk and nonlow-risk groups.

Combined model	Training cohort				Testing cohort				Independent-validation cohort			
	AUC (95%CI)	ACC %	SPE %	SEN %	AUC (95%CI)	ACC %	SPE %	SEN %	AUC (95%CI)	ACC %	SPE %	SEN %
Model_1	0.840 (0.812- 0.886)	73.1	73.1	76.6	0.826 (0.772- 0.865)	78.8	87.2	68.4	0.780 (0.742- 0.834)	70.9	78.0	67.0
Model_2	0.852 (0.820- 0.896)	74.1	74.1	70.5	0.845 (0.804- 0.876)	75.3	73.6	78.1	0.815 (0.773- 0.856)	73.1	76.0	71.4
Model_3	0.882 (0.846- 0.921)	80.7	80.7	82.7	0.882 (0.852- 0.931)	77.7	86.1	69.1	0.760 (0.722- 0.804)	70.2	70.0	70.3
Model_4	0.882 (0.860- 0.921)	77.2	77.2	81.4	0.878 (0.834- 0.917)	76.5	79.5	73.9	0.811 (0.763- 0.851)	71.6	84.0	64.8
Model_5	0.929 (0.875- 0.962)	85.3	85.3	84.5	0.917 (0.867- 0.940)	81.2	70.5	92.7	0.813 (0.768- 0.845)	73.1	72.0	73.6

Model_1, ADCintratumoral + T2intratumoral; Model_2, ADCintratumoral + T2peritumoral; Model_3, ADCperitumoral + T2intratumoral; Model_4, ADCperitumoral + T2peritumoral; Model_5, hybrid-feature (ADCintratumoral + ADCperitumoral + T2intratumoral + T2peritumoral); AUC, area under the curve; CI, confidence interval; ACC, accuracy; SPE, specificity; SEN, sensitivity.

### 3.5 Diagnostic performance of the radiomic nomogram

**Figure 4:**
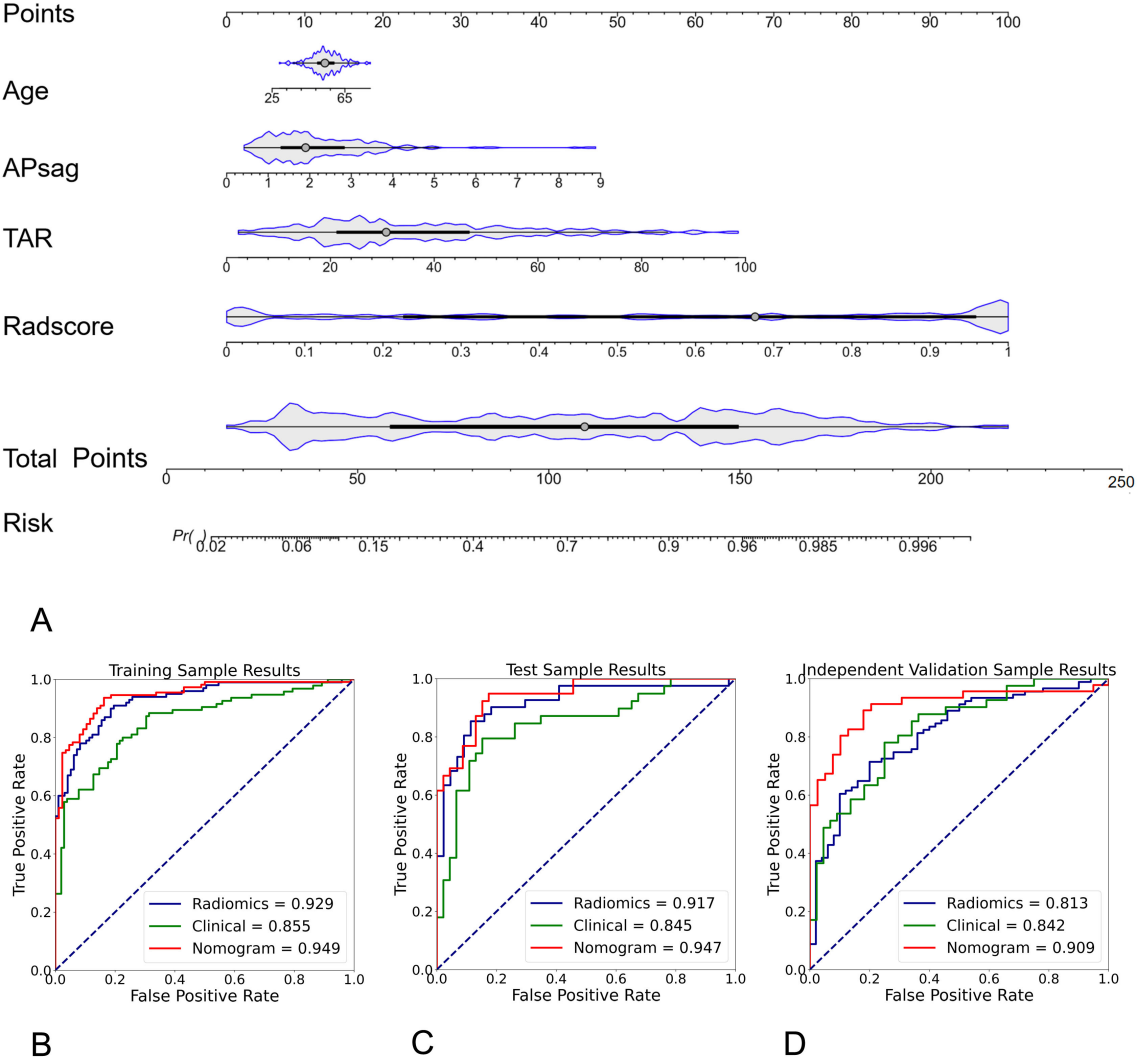
Radiomic nomogram for predicting low-risk and nonlow-risk EC. The nomogram was developed using morphological parameters (APsag and TAR), the radiomic signature (radscore), and patient age in the training cohort. A higher nomogram score indicates a greater likelihood of the patient having nonlow-risk EC. The formula for calculating the probability of a low-risk tumor is: 1 - Probability of a nonlow-risk tumor. Among all the models, the radiomic nomogram had the highest AUC of 0.949 in differentiating low-risk and nonlow-risk EC in the training **(B)**, test **(C)**, and independent validation cohort **(D)**.

To increase the predictive ability of the model, we combined the clinical and hybrid feature models to produce a clinical–radiomics mixed model. For the training cohort, a nomogram was constructed utilizing age, APsag, TAR, and the radscore (see **Figure 4A**). The AUC values of the nomogram model for classifying low-risk patients from the training (**Figure 4B**), test (**Figure 4C**), and independent validation (**Figure 4D**) cohorts were 0.949 (95% CI: 0.912, 0.970; sensitivity: 91.0%, specificity: 84.9%, accuracy: 88.3%, PPV:89.3%, NPV:86.4%), 0.947 (95% CI: 0.904, 0.962; sensitivity: 89.7%, specificity: 87.0%; accuracy: 88.2%, PPV:91.8%, NPV:83.3%), and 0.909 (95% CI: 0.876, 0.931; sensitivity: 82.6%, specificity: 87.2%; accuracy: 84.7%, PPV:90.1%, NPV:80.5%), respectively. The formula was as follows:.

Risk = -0.0172*Age + 0.498*APsag + 0.553*TAR + 2.092*Radscore.

**Figure 5:**
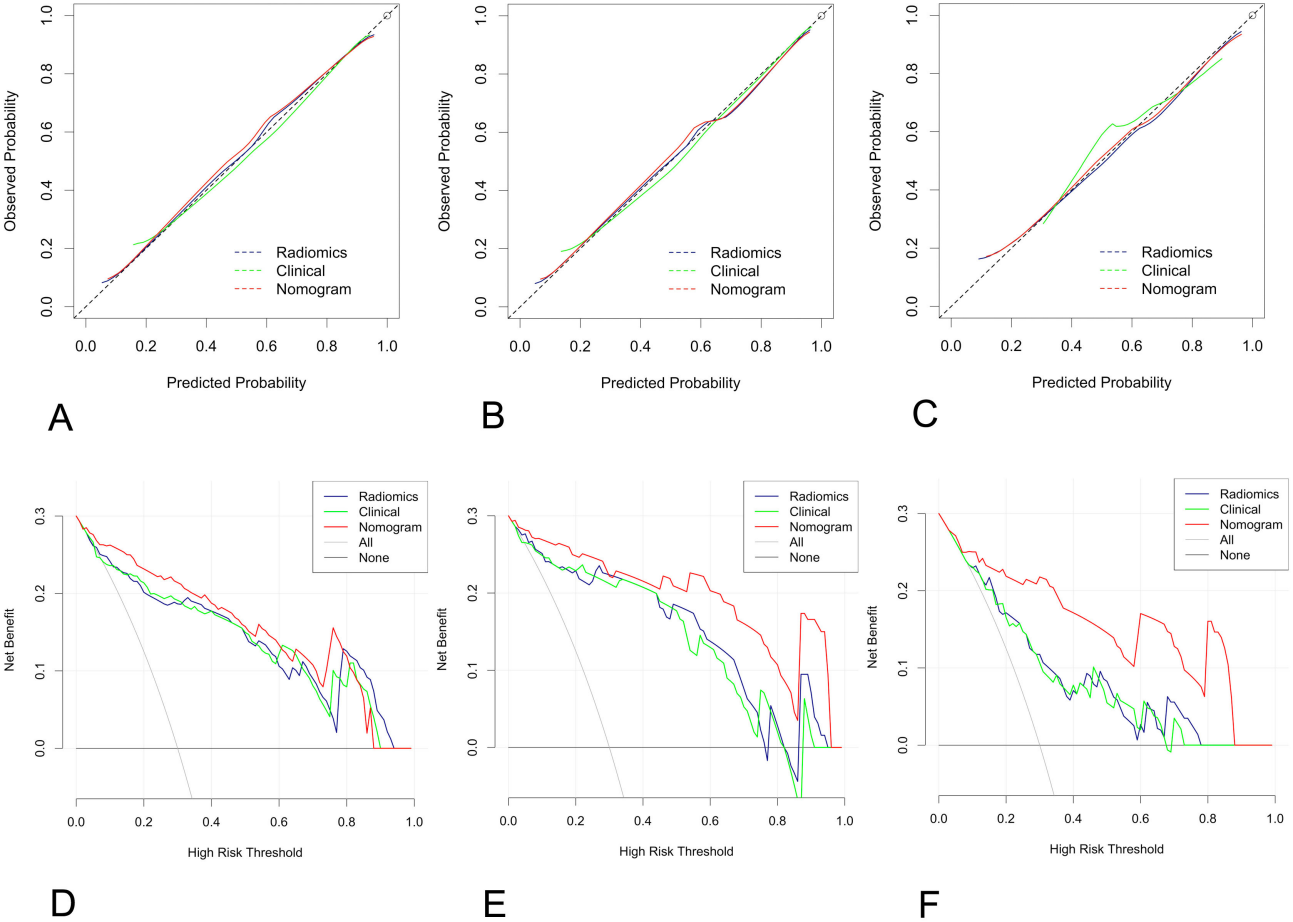
Calibration curve and decision curve analysis (DCA) plots of the nomogram. **(A-C)** Calibration curves of the nomogram in the training cohort **(A)**, the test cohort **(B)**, and the independent validation cohort **(C–F)** DCA plots. The vertical axis represents the net benefit, while the horizontal axis represents the threshold probability. The gray line represents the assumption that all patients are categorized as having nonlow-risk EC. The black line represents the assumption that none of the patients are considered to have nonlow-risk EC. The green line represents the output of the clinical model, the blue line represents the radiomic score, and the red line represents the output of the nomogram in the training **(D)**, test **(E)**, and independent validation cohorts **(F)**.

The nomogram calibration curve demonstrated good accuracy in predicting non-low-risk EC for the training, test and independent validation cohorts, as shown in **Figures 5A–C**. DCA confirmed the utility of the MRI radiomics nomogram for predicting non-low-risk EC in patients from both cohorts, as depicted in **Figures 5D–F**. The reclassification measures revealed that the nomogram outperformed both the radiomics and clinical models, with a net reclassification index (NRI) of 0.082 (95% CI, 0.048–0.134) in comparison with the radiomics model and an NRI of 0.164 (95% CI, 0.086–0.226) compared with the clinical model.

## 4 Discussion

In this study, we created and validated a simple radiomic nomogram for discriminating between low-risk and nonlow-risk EC according to age, APsag, TAR, and the radscore. The model demonstrated strong diagnostic accuracy (AUCtraining = 0.949, AUCtest = 0.947, AUCindependent-validation = 0.909). Additionally, the features extracted from different imaging sequences (specifically T2W images and ADC maps) and diverse VOIs (intratumoral, peritumoral, and combined regions) provided complementary data. Ultimately, a dataset was developed with diverse field strengths to act as an independent validation set, thus enhancing the models’ predictive performance in real-world situations.

The peritumoral region lies between the tumor and the surrounding healthy tissue (25). Despite appearing macroscopically similar to normal tissue, peritumoral tissue exhibits microscopic heterogeneity (26). In this study, combining intra- and peritumoral features proved to be valuable in predicting non-low-risk EC (ADC maps: AUCtrain = 0.850, AUCtest = 0.763, AUCindependent-validation = 0.719; T2W images: AUCtrain = 0.887, AUCtest = 0.827, AUCindependent-validation = 0.810). When we integrated the two imaging sequences for a range of VOIs, which included both intra- and peritumor regions, the hybrid features showed superior prediction efficiency (AUCtrain = 0.929, AUCtest = 0.917, AUCindependent validation = 0.813). This model incorporates key features such as the wavelet transform (WT), which includes histogram features (including kurtosis and skewness), texture features (such as the gray-level cooccurrence matrix (GLCM)), and shape features (specifically LeastAxisLength). The WT method is applied to separate images into high- and low-frequency components for both intratumoral and peritumoral areas (27). The GLCM is created by examining the connection between pairs of pixels and recording the occurrence of different combinations of gray levels in an image or region of interest. Compared with 2D ROIs, 3D VOIs have been shown to enhance the specificity of GLCM features in identifying tumor components (28). The GLCM was mentioned in a previous radiomic study on the risk classification of EC (15). Furthermore, a previous study revealed that kurtosis and skewness are correlated with the LVSI, DMI, and high-grade tumors of the EC (7). The original_shape_LeastAxisLength feature was derived from both ADC maps and T2WI image sequences concurrently, as highlighted in a prior study on LNM classification in EC (29). However, these indicators are essential for evaluating the risk stratification of tumors. Hence, extracting radiomic features from ADC maps and T2W images, both intra- and peritumoral, is essential for predicting non-low-risk EC patients.

In predicting non-low-risk EC patients, the clinical model with age, APsag, and TAR showed good performance, achieving AUC values of 0.855 for training, 0.845 for testing, and 0.842 for independent validation. The size of the tumor in EC is correlated with the extent of invasiveness, with larger tumors having a higher probability of DMI, LVSI, and LNM (30, 31). APsag and TAR are both indicators of TS in separate dimensions. TAR offers a simpler measurement approach by selecting the maximum cross section of the tumor. This method was proposed by Yan and colleagues (9). The results indicated that a TAR equal to or greater than 34.6% could lead to the accurate prediction of DMI in stage I EEC patients, with a sensitivity of 85.0% and a specificity of 84.8%. Furthermore, a TAR of 38.9% or higher was associated with the prediction of high-grade tumors, with a sensitivity of 83.3% and a specificity of 81.1%. A recent study comparing MRI-based texture analysis with APsag revealed that the latter was more efficient in predicting LVSI and high-grade EEC tumors prior to surgery (32). Furthermore, independent risk factors such as age, APsag, and TAR were found in a previous study that used MRI morphological parameters to be associated with predicting the risk stratification of EEC (23). The AUC results for predicting high-risk EEC using MRI morphological histograms, as highlighted by Yan et al., demonstrated consistency with both the training (1.5-T set, AUC = 0.856) and validation (3.0-T set, AUC = 0.849) cohorts. Our study yielded similar results, especially with the independent validation cohort, indicating that the clinical model performs reliably across datasets with different field strengths.

The nomogram constructed by combining age, APsag, TAR, and the radscore performed better that clinical models did in predicting non-low-risk EC, particularly in improving the robustness of the model (independent validation cohort, AUCclinical vs. AUCnomogram with 0.813 vs. 0.909). A recent meta-analysis further emphasized the importance of preoperative MRI radiomics models for risk stratification in patients with EC (33). Yan et al. (15) demonstrated the effectiveness of an MRI-based radiomic nomogram in predicting high-risk EC, with good performance for the validation groups, with AUCs ranging from 0.877-0.919. High-risk EC in their study was characterized by the presence of DMI, high-grade tumors, LVSI, CSI, LNM, non-EEC tumors, or EI. This classification corresponds with how patients in the non-low-risk group are classified according to ESMO guidelines. Despite not outperforming their model, our model requires fewer image sequences, which is advantageous for clinical applications. In comparison, their study depended on data from three distinct MRI sequences: T2W, diffusion-weighted, and contrast-enhanced T1W (CE-T1W) images. Considering the vital role of T2WI and DWI in the preoperative staging of EC patients and their potential cost-effectiveness over CE-T1WI, we suggest that radiomics models utilizing unenhanced images may offer more advantages and be easier to implement in clinical practice. Recently, a multiparametric MRI 3D radiomics-based machine learning model was developed and validated by Lefebvre TL et al. for predicting advanced International Federation of Gynecology and Obstetrics (FIGO) stage (IB or higher, considered non-low-risk by ESMO guidelines), achieving a test set performance of 0.84 (34). More recently, Lin et al. (35) developed RadSignature, which incorporates age, tumor type, size, and grade. This model achieved moderate performance (with an accuracy of 75.4%) in predicting high-risk patients with a test set. Compared with previous radiomic and ML models, our model demonstrated improved diagnostic performance with the validation cohort, with an AUC of 0.909.

This study offers numerous potential advantages. (1) The prediction model exhibits excellent robustness when tested with cross-field strength datasets. Various field strengths, manufacturers, and scanning protocols present challenges for artificial intelligence prediction models. Our prediction model was developed using a 1.5-T dataset, and it has shown outstanding predictive performance when tested using the same field strength dataset. This model was also independently validated with a 3.0-T dataset, demonstrating the model’s strong generalizability and its potential as a valuable tool for handling real-world problems. (2) The complementary information provided by intra- and peritumoral radiomic features offers a new strategy for developing AI models to predict the risk stratification of EC. To our knowledge, this MRI radiomics model is the first to incorporate peritumoral features in the prediction of ESMO risk stratification of EC. (3) Our nomogram will prove valuable for patients with endometrioid grades 1–2 histology at preoperative biopsy who are deemed non-low-risk EEC, provided that lymphadenectomy is a feasible option. This is particularly relevant in cases where sentinel node procedures are unsuccessful (observed in 4%-22% of cases depending on the surgeon’s skill level and technical challenges) (36) or in facilities where this technique cannot currently be implemented.

Our study had several limitations. First, this study was a retrospective single-center study, even though we established an independent validation cohort to assess the strength of this model. Moving forward, we plan to conduct multicenter prospective research to confirm the generalizability of our findings. Second, manual whole-tumor segmentation was carried out instead of relying on automatic or semiautomatic methods. These manual segmentations could introduce subjectivity and result in bias, even though we assessed interreader agreement. Our goal in future studies will involve designing an automatic network based on deep learning to minimize tumor segmentation variability and improve segmentation efficiency. Third, advanced risk stratification approaches that include additional features, such as the EC molecular profile, could be utilized in categorizing patients (37, 38). Nevertheless, their usage is not prevalent, and the lack of data calls for further, more extensive research in the future. Moreover, the model is constructed solely on T2W images and ADC maps, omitting the consideration of contrast-enhanced T1W images, which could result in the oversight of important data. However, our study included an independent validation cohort, reducing the likelihood of overfitting. Finally, patients who did not receive lymphadenectomy were classified as negative for LNM, potentially creating bias, even though the surgeons made efforts to evaluate lymph node status during the procedure.

In conclusion, we developed and validated an MRI radiomics nomogram model by combining age, APsag, TAR and the radscore. Owing to its good diagnostic performance, this nomogram can effectively distinguish between low-risk and non-low-risk groups, suggesting potential clinical usefulness for surgical management in patients with EC. However, more studies are necessary to validate its performance in this domain, ideally through a prospective trial format.

## Data Availability

The datasets generated for this study are available on request to the first author. Requests to access these datasets should be directed to Bin Yan, yanbin3t2008@yahoo.com.
