# Supplementary material for: Intratumoral and peritumoral multiparametric MRI-based radiomics nomogram for preoperative risk stratification in patients with endometrial cancer
Source: Front Oncol. 2025 Aug 26;15:1572784. doi: 10.3389/fonc.2025.1572784 (PMC12418119; doi:10.3389/fonc.2025.1572784)
Supplement: Supplementary file 1 [file DataSheet1.docx]

Supplementary Material

# 2 Materials and methods

**2.3 MRI protocol and tumor segmentation**

Supplementary Table 1. Scanning sequence and parameters of the 1.5-T and 3.0-T MR scanners

| **Pulse Sequence** | **1.5-T MR** | **3.0-T MR** |
| --- | --- | --- |
| Axial T2WI | FRFSE, TR/TE = 4450/90 msec; matrix = 336 × 224; FOV = 280 × 400 mm; NEX = 2;  section thickness/intersection gap = 5/1.5 mm | TSE, TR/TE = 4290/93 msec; matrix = 384 × 345; FOV = 280×400 mm; NEX = 2;  section thickness/intersection gap = 5/1 mm |
| Axial T1WI | FSE, TR/TE = 605/15 msec; matrix = 336 × 224; FOV = 280 × 400 mm; NEX = 2;  section thickness/intersection gap = 5/1.5 mm | Dixon-VIBE, TR/TE = 5.55 /2.46 msec; matrix = 320 × 272; FOV = 280 × 400 mm; NEX = 1; section thickness/intersection gap = 4 /0.4 mm |
| Axial DWI | EPI, TR/TE/TI = 6900/80/150 ms;  b factors = 0 and 650 s/mm^2^; matrix = 128 × 128; FOV=280 × 400 mm; section thickness/intersection gap = 5/1.5 mm; speeder flag = 2; NEX = 4 | RESOLVE DWI, TR/TE = 5340/62 ms;  b factors = 0 and 1000 s/mm^2^;  matrix = 160 × 160; FOV = 280 × 400 mm;  section thickness/intersection gap = 5/1 mm; NEX = 3; readout segments = 5;  readout partial Fourier = 5/8. |
| Obligue T2WI | FRFSE, TR/TE = 2629/80 msec; matrix = 256 × 192; FOV = 250 × 200 mm; NEX = 2;  section thickness/intersection gap = 3.5/0.5 mm | TSE, TR/TE = 4750/108 msec; matrix = 320 × 224; FOV = 200 × 200 mm; NEX = 2;  section thickness/intersection gap = 3 /0.6 mm |
| Sagittal T2WI | FRFSE, TR/TE = 3324/90 msec; matrix = 320 × 224; FOV = 250 × 230 mm; NEX = 2;  section thickness/intersection gap = 4/1 mm | TSE, TR/TE = 6100 / 91 msec; matrix = 320 × 262; FOV = 250 × 230 mm; NEX = 2;  section thickness/intersection gap = 4 /0.4 mm |

DWI, diffusion-weighted imaging; EPI, echo-planar imaging; FSE, fast spin‒echo; FRFSE, fast-recovery fast spin‒echo; FOV, field of view; RESOLVE, readout segmentation of long variable echo-trains; TR, repetition time; TE, echo time; TI, inversion time; NEX, number of excitations; VIBE, volumetric interpolated breath-hold examination

**2.4 Radiomic features**

**Image preprocessing** was carried out via a standard workflow. Prior to feature extraction, it was essential to standardize the images. This includes resampling the spacing of all images to a consistent scale of [1.0, 1.0, 1.0], normalizing the grayscale intensity across all images via Z score normalization techniques, and applying Gaussian filtering to decrease noise in the images. Ensuring the homogeneity and comparability of the dataset through these methodological procedures is vital, as it greatly increases the accuracy and reliability of the subsequent feature extraction steps.

**Image segmentation** was carried out independently by two radiologists specializing in gynecological oncology MRI: reader_1 (T.Z.) with 5 years of experience and reader_2 (B.Y.) with 10 years of experience. Reader_2 confirmed all the tumor segmentations. In cases where the two readers agreed, the segmentation was finalized. If there were differing opinions, the final segmentation was determined through discussion. All confirmed regions of interest (ROIs) were included in the analysis (reader_confirmed). Additionally, to evaluate interreader reliability, the images of 100 patients were randomly selected one month later for tumor segmentation by a third radiologist, reader_3 (Y.D.), with 8 years of experience in gynecological oncology MRI.

**3 Results**

**3.2 Radiomics feature extraction, selection, and interreader reliability**

The original feature data were filtered with the independent-sample t test and correlation testing. Features with P > 0.05 or a correlation coefficient > 0.9 were excluded from the original data. After these features were removed, the number of features in the original data was reduced from 1036 to 136, 90, 93, and 73 in the ADCintratumoral, ADCperitumoral, T2intratumoral and T2peritumoral feature groups, respectively.

To retain the most discriminative features from the filtered data, the least absolute shrinkage and selection operator (LASSO) was used. LASSO is the most appropriate method for searching for the most rewarding features in high-dimensional data [1]. After LASSO regression, the number of features decreased from 136, 90, 93 and 73 to 5, 7, 7 and 6 in the ADCintratumoral, ADCperitumoral, T2_intratumoral and T2peritumoral feature sets, respectively (detailed in Supplementary Table 2).

To establish a combined single-sequence intratumoral and peritumoral model, 5 ADCintratumoral features and 7 ADCperitumoral features were selected to comprise the 12 ADC features, and 7 T2intratumoral features and 6 T2peritumoral features were selected to comprise the 13 T2 features. After LASSO regression, the number of ADC features was reduced from 12 to 9, and the number of T2 features was reduced from 13 to 9 (detailed in Supplementary Table 3). These six groups of features were subsequently used to construct six logistic regression (LR) models, and all the features had nonzero coefficients.

To establish the combined ADC-map and T2WI-feature models, the 5 ADCintratumoral and 7 T2WIintratumoral features were combined to establish a 12-feature set for creating Model_1; the 5 ADCintratumoral and 6 T2WIperitumoral features were combined to create an 11-feature set for establishing Model_2; the 7 ADCperitumoral and 7 T2intratumoral features were combined to create a 14-feature set for establishing Model_3; and the 7 ADCperitumoral and 6 T2peritumoral features were combined to create a 13-feature set for establishing Model_4. After LASSO regression, the number of features for constructing Model_1, Model_2, Model_3, and Model_4 were reduced from 12, 11, 14, and 13 to 8, 6, 7 and 8, respectively (detailed in Supplementary Table 3).

All 25 VOI features (ADCintratumoral + ADCperitumoral + T2WIintratumoral + T2WIperitumoral) were combined and used to create the hybrid model (Model_5). Combining the ADC map and T2WI, 18 features were included: 9 were from the ADC maps, and 9 were from T2WI. Finally, LASSO was utilized to select the most valuable features. After LASSO filtering, the coefficients of the 9 features were 0, so the remaining 9 features were ultimately retained in the hybrid model.

Supplementary Table 2 Radiomics feature selection procedures for each VOI and prediction model construction

| **Model** | **Features** | **Number** |
| --- | --- | --- |
| ADCintratumoral | 'original_shape_LeastAxisLength',  'wavelet-HHH_firstorder_Skewness',  'wavelet-LLL_glcm_MCC',  'wavelet-LHH_glszm_GrayLevelNonUniformityNormalized',  'original_glcm_Imc1' | 5 |
| ADCperitumoral | 'wavelet-LHL_firstorder_Minimum',  'log-sigma-5-0-mm-3D_glcm_Imc2',  'log-sigma-5-0-mm-3D_glcm_ClusterShade',  'wavelet-LLH_glcm_MCC',  'original_shape_LeastAxisLength',  'wavelet-HHL_glcm_ClusterShade',  'log-sigma-3-0-mm-3D_firstorder_90Percentile' | 7 |
| T2intratumoral | 'original_glcm_Contrast',  'wavelet-HLH_firstorder_Skewness',  'log-sigma-5-0-mm-3D_glcm_Imc1',  'original_firstorder_Kurtosis',  'original_shape_LeastAxisLength',  'wavelet-HLL_glrlm_LowGrayLevelRunEmphasis',  'wavelet-LLH_glrlm_LongRunLowGrayLevelEmphasis' | 7 |
| T2peritumoral | 'original_shape_LeastAxisLength',  'original_glcm_ClusterShade',  'original_gldm_LargeDependenceHighGrayLevelEmphasis',  'log-sigma-3-0-mm-3D_glrlm_LowGrayLevelRunEmphasis',  'log-sigma-5-0-mm-3D_glcm_Correlation',  'wavelet-LHH_glrlm_LongRunEmphasis' | 6 |

Supplementary Table 3. Combined features for the tumor feature experiment and prediction model construction

| **Model** | **Features** | **Number** | **Model** |
| --- | --- | --- | --- |
| ADCintratumoral+ peritumoral | 'Intratumoral_original_shape_LeastAxisLength',  'Intratumoral_wavelet-HHH_firstorder_Skewness',  'Intratumoral_original_glcm_Imc1',  'Peritumoral_wavelet-LHL_firstorder_Minimum',  'Peritumoral_log-sigma-5-0-mm-3D_glcm_Imc2',  'Peritumoral_log-sigma-5-0-mm-3D_glcm_ClusterShade'  Peritumoral_wavelet-LLH_glcm_MCC'  Peritumoral_original_shape_LeastAxisLength'  Peritumoral_wavelet-HHL_glcm_ClusterShade' | 9 | LR |
| T2intratumoral+ peritumoral | 'Intratumoral_original_glcm_Contrast',  'Intratumoral_wavelet-HHL_firstorder_Kurtosis',  'Intratumoral_wavelet-HLH_firstorder_Skewness',  'Intratumoral_log-sigma-5-0-mm-3D_glcm_Imc1',  'Intratumoral_original_firstorder_Kurtosis',  'Intratumoral_original_shape_LeastAxisLength',  'Peritumoral_original_shape_LeastAxisLength',  'Peritumoral_original_glcm_ClusterShade',  'Peritumoral_log-sigma-5-0-mm-3D_glcm_Correlation', | 9 | LR |
| Model_1:  ADCintratumoral +  T2WIintratumoral | 'ADCintratumoral_original_shape_LeastAxisLength',  'ADCintratumoral_wavelet-HHH_firstorder_Skewness',  'ADCintratumoral_wavelet-LLL_glcm_MCC',  'T2intratumoral_wavelet-HHL_firstorder_Kurtosis',  'T2intratumoral_wavelet-HLH_firstorder_Skewness',  'T2intratumoral_log-sigma-5-0-mm-3D_glcm_Imc1',  'T2intratumoral_original_firstorder_Kurtosis',  'T2intratumoral_original_shape_LeastAxisLength' | 8 | LR |
| Model_2:  ADCintratumoral +  T2WIperitumoral | 'ADCintratumoral_original_shape_LeastAxisLength',  'ADCintratumoral_wavelet-HHH_firstorder_Skewness',  'ADCintratumoral_wavelet-LLL_glcm_MCC',  'T2peritumoral_original_shape_LeastAxisLength',  'T2peritumoral_original_glcm_ClusterShade',  'T2peritumoral_original_gldm_LargeDependenceHighGrayLevelEmphasis' | 6 | LR |
| Model_3:  ADCperitumoral +  T2WIintratumoral | 'ADCperitumoral_wavelet-LLH_glcm_MCC',  'ADCperitumoral_original_shape_LeastAxisLength',  'ADCperitumoral_wavelet-HHL_glcm_ClusterShade',  'ADCperitumoral_wavelet-LHL_firstorder_Minimum',  'T2intratumoral_original_firstorder_Kurtosis',  'T2intratumoral_original_shape_LeastAxisLength',  'T2intratumoral_wavelet-HLL_glrlm_LowGrayLevelRunEmphasis' | 7 | LR |
| Model_4:  ADCperitumoral +  T2WIperitumoral | 'ADCperitumoral _log-sigma-5-0-mm-3D_glcm_ClusterShade',  'ADCperitumoral _wavelet-LLH_glcm_MCC',  'ADCperitumoral _original_shape_LeastAxisLength',  'ADCperitumoral _wavelet-HHL_glcm_ClusterShade',  'ADCperitumoral _log-sigma-3-0-mm-3D_firstorder_90Percentile'  'T2peritumoral _original_shape_LeastAxisLength',  'T2peritumoral _original_glcm_ClusterShade'  'T2peritumoral _log-sigma-5-0-mm-3D_glcm_Correlation', | 8 | LR |
| Model_5(hybrid-feature):  ADCintratumoral+  ADCperitumoral+  T2intratumoral+  T2peritumoral | 'ADCintratumoral_original_shape_LeastAxisLength',  'ADCintratumoral_wavelet-HHH_firstorder_Skewness',  'ADCperitumoral_wavelet-LLH_glcm_MCC',  'ADCperitumoral_original_shape_LeastAxisLength',  'T2intratumoral_original_glcm_Contrast',  'T2intratumoral_wavelet-HHL_firstorder_Kurtosis',  'T2intratumoral_wavelet-HLH_firstorder_Skewness',  'T2peritumoral_original_shape_LeastAxisLength',  'T2peritumoral_original_glcm_ClusterShade' | 9 | LR |

LR, logistic regression

Interobserver reliability

Excellent interreader reliability was obtained for all the morphological parameters and radiomic features (ICC = 0.902‒0.997). The details are shown in Supplementary Tables 4-6.

Supplementary Table 4. Interreader variability of tumor measurements performed by three readers for the data of 100 patients

| MRI morphological parameter | 1.5T-MR cohort (n=65) | 3.0T-MR cohort (n=35) |
| --- | --- | --- |
|  | ICC (95% CI) | ICC (95% CI) |
| Tumor volume (cm^3^) | 0.997 (0.995-0.998) | 0.973 (0.943-0.987) |
| APsag (cm) | 0.956 (0.925-0.973) | 0.943 (0.885-0.973) |
| Tumor size (cm) | 0.983 (0.971-0.989) | 0.934 (0.866-0.968) |
| Area of tumor (cm^2^) | 0.983 (0.970-0.989) | 0.969 (0.934-0.985) |
| Area of uterus (cm^2^) | 0.992 (0.988-0.996) | 0.961 (0.919-0.981) |

APsag, maximum anteroposterior tumor diameter on sagittal T2-weighted imaging.

Supplementary Table 5. Interreader variability of radiomic features (intratumoral region) measured on ADC maps by three readers for the data of 100 patients

| Radiomics feature (ADC mapping) | 1.5T-MR cohort (n=65) | 3.0T-MR cohort (n=35) |
| --- | --- | --- |
|  | ICC | ICC |
| original_shape_LeastAxisLength (ADCintratumoral) | 0.923(0.889-0.945) | 0.936(0.904-0.952) |
| wavelet-HHH_firstorder_Skewness (ADCintratumoral) | 0.995(0.979-0.998) | 0.906(0.885-0.919) |
| wavelet-LLL_glcm_MCC (ADCintratumoral) | 0.912(0.875-0.929) | 0.941(0.903-0.965) |
| wavelet-LHH_glszm_GrayLevelNonUniformityNormalized  (ADCintratumoral) | 0.959(0.938-0.986) | 0.950(0.934-0.970) |
| original_glcm_Imc1 (ADCintratumoral) | 0.902(0.881-0.921) | 0.946(0.925-0.966) |

Supplementary Table 6. Interreader variability of radiomic features (intratumoral region) measured on T2W images by three readers for the data of 100 patients

| Radiomics feature (T2 mapping) | 1.5T-MR cohort (n=65) | 3.0T-MR cohort (n=35) |
| --- | --- | --- |
|  | ICC | ICC |
| original_glcm_Contrast (T2intratumoral) | 0.940(0.919-0.959) | 0.963(0.938-0.972) |
| wavelet-HLH_firstorder_Skewness (T2intratumoral) | 0.935(0.923-0.952) | 0.921(0.903-0.935) |
| log-sigma-5-0-mm-3D_glcm_Imc1 (T2intratumoral) | 0.993(0.983-0.999) | 0.962(0.943-0.982) |
| original_firstorder_Kurtosis (T2intratumoral) | 0.991(0.979-0.996) | 0.959(0.935-0.981) |
| original_shape_LeastAxisLength (T2intratumoral) | 0.942(0.922-0.960) | 0.965(0.943-0.973) |
| wavelet-HLL_glrlm_LowGrayLevelRunEmphasis (T2intratumoral) | 0.935(0.926-0.965) | 0.938(0.901-0.955) |
| wavelet-LLH_glrlm_LongRunLowGrayLevelEmphasis (T2intratumoral) | 0.982(0.961-0.992) | 0.961(0.919-0.985) |

**References:**

[1] Tibshirani R. Regression Shrinkage and Selection via the Lasso. Journal of the Royal Statistical Society, Series B (Methodological) 1996;73:267-288. <http://citeseerx.ist.psu.edu/viewdoc/download;jsessionid=005D6C59BC707829AEB52FC61BDD6C6D?doi=10.1.1.35.7574&rep=rep1&type=pdf>.
